# 5,5′-Dimeth­oxy-2,2′-[4,5-dimethyl-*o*-phenyl­enebis(nitrilo­methyl­idyne)]diphenol

**DOI:** 10.1107/S1600536810007282

**Published:** 2010-03-03

**Authors:** Hadi Kargar, Reza Kia, Islam Ullah Khan, Atefeh Sahraei, Parviz Aberoomand Azar

**Affiliations:** a Department of Chemistry, School of Science, Payame Noor University (PNU), Ardakan, Yazd, Iran; b Department of Chemistry, Science and Research Branch, Islamic Azad University, Tehran, Iran; c Materials Chemistry Laboratory, Department of Chemistry, GC University, Lahore 54000, Pakistan

## Abstract

In the crystal structure of the title compound, C_24_H_24_N_2_O_4_, the dihedral angles between the central and the two outer benzene rings are 48.12 (8) and 21.44 (8)°. Intra­molecular O—H⋯N hydrogen bonding generates *S*(6) rings.

## Related literature

For graph-set notation see: Bernstein *et al.* (1995 ▶). For a related structure see: Kargar *et al.* (2010 ▶).
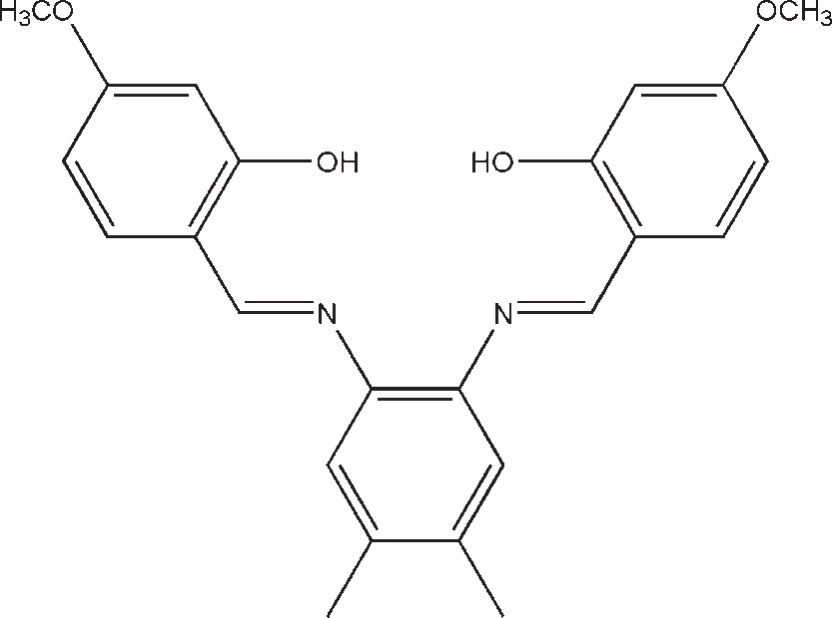


## Experimental

### 

#### Crystal data

C_24_H_24_N_2_O_4_
*M* *_r_* = 404.45
Monoclinic, 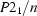

*a* = 6.6720 (3) Å
*b* = 14.3192 (6) Å
*c* = 21.7396 (9) Å
β = 95.177 (2)°
*V* = 2068.48 (15) Å^3^
*Z* = 4
Mo *K*α radiation
μ = 0.09 mm^−1^
*T* = 296 K
0.42 × 0.32 × 0.18 mm

#### Data collection

Bruker SMART APEXII CCD area-detector diffractometer
Absorption correction: multi-scan (*SADABS*; Bruker, 2005 ▶) *T*_min_ = 0.964, *T*_max_ = 0.984
23605 measured reflections
5123 independent reflections
3112 reflections with *I* > 2σ(*I*)
*R*_int_ = 0.034

#### Refinement

*R*[*F* ^2^ > 2σ(*F* ^2^)] = 0.046
*wR*(*F* ^2^) = 0.125
*S* = 1.01
5123 reflections
274 parameters
H-atom parameters constrained
Δρ_max_ = 0.15 e Å^−3^
Δρ_min_ = −0.16 e Å^−3^

### 

Data collection: *APEX2* (Bruker, 2005 ▶); cell refinement: *SAINT* (Bruker, 2005 ▶); data reduction: *SAINT*; program(s) used to solve structure: *SHELXS97* (Sheldrick, 2008 ▶); program(s) used to refine structure: *SHELXL97* (Sheldrick, 2008 ▶); molecular graphics: *SHELXTL* (Sheldrick, 2008 ▶); software used to prepare material for publication: *SHELXTL* and *PLATON* (Spek, 2009 ▶).

## Supplementary Material

Crystal structure: contains datablocks global, I. DOI: 10.1107/S1600536810007282/nc2177sup1.cif

Structure factors: contains datablocks I. DOI: 10.1107/S1600536810007282/nc2177Isup2.hkl

Additional supplementary materials:  crystallographic information; 3D view; checkCIF report

## Figures and Tables

**Table 1:** Hydrogen-bond geometry (Å, °)

*D*—H⋯*A*	*D*—H	H⋯*A*	*D*⋯*A*	*D*—H⋯*A*
O1—H1⋯N1	0.95	1.78	2.6158 (18)	145
O2—H2⋯N2	0.96	1.71	2.5791 (17)	150

**Fig. 1.**
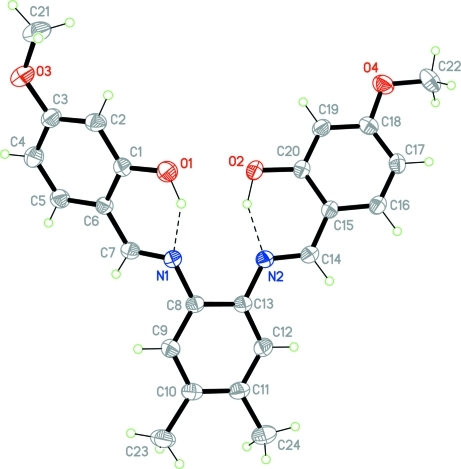
Crystal structure of the title compound, showing 30% probability displacement ellipsoids and the atomic numbering. Intramolecular hydrogen bonds are drawn as dashed lines.

## supplementary crystallographic information

### Comment

The crystal structure of the title compound was determined to clarify the identity of the synthetic product and to compare the structural changes upon complexation with Cu(II) and Ni(II) ions in future works.

In the crystal structure of the title compound the dihedral angles between the central benzene ring and the two outer benzene rings amount to 48.12 (8) and 21.44 (8)°.  The bond lengths and angles are comparable to those in a related structure reported recently (Kargar *et al.,* 2010). Intramolecular O—H···N hydrogen bonding is found that generates six-membered *S(6)* rings. (Bernstein *et al.,* 1995) (Table 1).

### Experimental

The title compound was synthesized by adding 4-methoxy-salicylaldehyde (4 mmol) to a solution of 4,5-dimethyl-*o*-phenylenediamine (2 mmol) in ethanol (20 ml). The mixture was refluxed with stirring for half an hour. The resultant yellow solution was filtered. Yellow single crystals of the title compound suitable for *X*-ray structure determination were recrystallized from ethanol by slow evaporation of the solvents at room temperature over several days.

### Refinement

The position of the H atoms of the hydroxy groups were located in a difference Fourier map but finally they were refined using a riding model with U_iso_(H) = 1.5 U_eq_(O). The remaining H atoms were positioned with idealized geometry (methyl H atoms allowed to rotate but not to tip) with C-H = 0.93-0.96 Å and refined using a riding model with U_iso_(H) = 1.2 or 1.5 U_eq_ (C).

### Crystal data

**Table:**

C_24_H_24_N_2_O_4_	*F*(000) = 856
*M**_r_* = 404.45	*D*_x_ = 1.299 Mg m^−^^3^
Monoclinic, *P*2_1_/*n*	Mo *K*α radiation, λ = 0.71073 Å
Hall symbol: -P 2yn	Cell parameters from 5431 reflections
*a* = 6.6720 (3) Å	θ = 2.4–26.4°
*b* = 14.3192 (6) Å	µ = 0.09 mm^−^^1^
*c* = 21.7396 (9) Å	*T* = 296 K
β = 95.177 (2)°	Block, yellow
*V* = 2068.48 (15) Å^3^	0.42 × 0.32 × 0.18 mm
*Z* = 4	

### Data collection

**Table:**

Bruker SMART APEXII CCD area-detector diffractometer	5123 independent reflections
Radiation source: fine-focus sealed tube	3112 reflections with *I* > 2σ(*I*)
graphite	*R*_int_ = 0.034
φ and ω scans	θ_max_ = 28.3°, θ_min_ = 1.7°
Absorption correction: multi-scan (*SADABS*; Bruker, 2005)	*h* = −8→6
*T*_min_ = 0.964, *T*_max_ = 0.984	*k* = −19→13
23605 measured reflections	*l* = −28→28

### Refinement

**Table:**

Refinement on *F*^2^	Primary atom site location: structure-invariant direct methods
Least-squares matrix: full	Secondary atom site location: difference Fourier map
*R*[*F*^2^ > 2σ(*F*^2^)] = 0.046	Hydrogen site location: inferred from neighbouring sites
*wR*(*F*^2^) = 0.125	H-atom parameters constrained
*S* = 1.01	*w* = 1/[σ^2^(*F*_o_^2^) + (0.0473*P*)^2^ + 0.4824*P*] where *P* = (*F*_o_^2^ + 2*F*_c_^2^)/3
5123 reflections	(Δ/σ)_max_ = 0.001
274 parameters	Δρ_max_ = 0.15 e Å^−^^3^
0 restraints	Δρ_min_ = −0.16 e Å^−^^3^

### Special details

**Table:**

Geometry. All esds (except the esd in the dihedral angle between two l.s. planes) are estimated using the full covariance matrix. The cell esds are taken into account individually in the estimation of esds in distances, angles and torsion angles; correlations between esds in cell parameters are only used when they are defined by crystal symmetry. An approximate (isotropic) treatment of cell esds is used for estimating esds involving l.s. planes.
Refinement. Refinement of F^2^ against ALL reflections. The weighted R-factor wR and goodness of fit S are based on F^2^, conventional R-factors R are based on F, with F set to zero for negative F^2^. The threshold expression of F^2^ > 2sigma(F^2^) is used only for calculating R-factors(gt) etc. and is not relevant to the choice of reflections for refinement. R-factors based on F^2^ are statistically about twice as large as those based on F, and R- factors based on ALL data will be even larger.

### Fractional atomic coordinates and isotropic or equivalent isotropic displacement parameters (Å^2^)

**Table:**

	*x*	*y*	*z*	*U*_iso_*/*U*_eq_	
O1	0.80563 (17)	1.05843 (9)	0.32895 (6)	0.0605 (4)	
H1	0.8156	1.0315	0.2894	0.091*	
O2	0.61776 (18)	0.82964 (9)	0.31447 (5)	0.0601 (4)	
H2	0.6865	0.8448	0.2791	0.090*	
O3	1.2169 (2)	1.11971 (10)	0.51656 (6)	0.0720 (4)	
O4	0.03885 (19)	0.66990 (9)	0.35951 (6)	0.0634 (4)	
N1	0.9786 (2)	0.97538 (9)	0.24052 (6)	0.0424 (3)	
N2	0.6943 (2)	0.84762 (9)	0.20089 (6)	0.0437 (3)	
C1	0.9858 (2)	1.05519 (11)	0.36245 (7)	0.0423 (4)	
C2	1.0013 (3)	1.09115 (12)	0.42227 (8)	0.0500 (4)	
H2A	0.8890	1.1163	0.4386	0.060*	
C3	1.1851 (3)	1.08903 (12)	0.45710 (8)	0.0517 (4)	
C4	1.3543 (3)	1.05316 (14)	0.43257 (8)	0.0579 (5)	
H4A	1.4783	1.0535	0.4558	0.070*	
C5	1.3372 (3)	1.01751 (13)	0.37431 (8)	0.0516 (4)	
H5A	1.4507	0.9926	0.3585	0.062*	
C6	1.1544 (2)	1.01719 (11)	0.33750 (7)	0.0406 (4)	
C7	1.1429 (2)	0.98008 (11)	0.27563 (7)	0.0423 (4)	
H7A	1.2605	0.9585	0.2605	0.051*	
C8	0.9810 (2)	0.93897 (11)	0.18012 (7)	0.0405 (4)	
C9	1.1249 (2)	0.96510 (12)	0.14096 (7)	0.0469 (4)	
H9A	1.2213	1.0091	0.1546	0.056*	
C10	1.1297 (3)	0.92783 (13)	0.08221 (7)	0.0500 (4)	
C11	0.9840 (3)	0.86310 (13)	0.06121 (8)	0.0513 (4)	
C12	0.8381 (3)	0.83831 (12)	0.09976 (8)	0.0499 (4)	
H12A	0.7403	0.7952	0.0856	0.060*	
C13	0.8323 (2)	0.87538 (11)	0.15879 (7)	0.0411 (4)	
C14	0.5294 (2)	0.80505 (11)	0.18428 (7)	0.0437 (4)	
H14A	0.4933	0.7952	0.1425	0.052*	
C15	0.3988 (2)	0.77214 (11)	0.22869 (7)	0.0396 (4)	
C16	0.2190 (2)	0.72666 (12)	0.20999 (8)	0.0468 (4)	
H16A	0.1827	0.7193	0.1680	0.056*	
C17	0.0929 (3)	0.69218 (12)	0.25145 (8)	0.0498 (4)	
H17A	−0.0263	0.6620	0.2377	0.060*	
C18	0.1477 (3)	0.70345 (11)	0.31392 (8)	0.0468 (4)	
C19	0.3228 (3)	0.75016 (12)	0.33444 (8)	0.0491 (4)	
H19A	0.3560	0.7585	0.3766	0.059*	
C20	0.4481 (2)	0.78427 (11)	0.29270 (7)	0.0430 (4)	
C21	1.0541 (4)	1.15847 (16)	0.54511 (10)	0.0783 (7)	
H21A	1.0992	1.1773	0.5864	0.117*	
H21B	0.9495	1.1127	0.5463	0.117*	
H21C	1.0031	1.2118	0.5220	0.117*	
C22	−0.1404 (3)	0.61818 (16)	0.34162 (11)	0.0744 (6)	
H22A	−0.2026	0.5996	0.3778	0.112*	
H22B	−0.1069	0.5637	0.3189	0.112*	
H22C	−0.2319	0.6565	0.3161	0.112*	
C23	1.2948 (3)	0.95713 (16)	0.04274 (9)	0.0708 (6)	
H23A	1.2358	0.9807	0.0039	0.106*	
H23B	1.3781	0.9042	0.0356	0.106*	
H23C	1.3753	1.0050	0.0637	0.106*	
C24	0.9800 (3)	0.81878 (17)	−0.00213 (9)	0.0763 (6)	
H24A	0.8676	0.7769	−0.0081	0.114*	
H24B	1.1026	0.7847	−0.0053	0.114*	
H24C	0.9673	0.8666	−0.0332	0.114*	

### Atomic displacement parameters (Å^2^)

**Table:**

	*U*^11^	*U*^22^	*U*^33^	*U*^12^	*U*^13^	*U*^23^
O1	0.0412 (7)	0.0832 (9)	0.0572 (8)	0.0083 (6)	0.0045 (6)	−0.0120 (6)
O2	0.0573 (8)	0.0784 (9)	0.0436 (7)	−0.0228 (7)	−0.0005 (6)	−0.0057 (6)
O3	0.0826 (10)	0.0852 (10)	0.0475 (7)	−0.0034 (8)	0.0031 (7)	−0.0143 (7)
O4	0.0621 (8)	0.0663 (8)	0.0646 (8)	−0.0111 (7)	0.0203 (7)	0.0011 (6)
N1	0.0423 (8)	0.0461 (8)	0.0390 (7)	−0.0018 (6)	0.0049 (6)	−0.0045 (6)
N2	0.0458 (8)	0.0452 (8)	0.0405 (7)	−0.0030 (6)	0.0063 (6)	−0.0035 (6)
C1	0.0393 (9)	0.0436 (9)	0.0439 (9)	−0.0004 (7)	0.0037 (7)	0.0004 (7)
C2	0.0556 (11)	0.0462 (9)	0.0501 (10)	0.0032 (8)	0.0155 (8)	−0.0041 (8)
C3	0.0633 (12)	0.0514 (10)	0.0398 (9)	−0.0063 (9)	0.0019 (8)	−0.0017 (8)
C4	0.0498 (11)	0.0734 (13)	0.0491 (10)	−0.0007 (9)	−0.0045 (8)	0.0008 (9)
C5	0.0442 (10)	0.0628 (11)	0.0480 (10)	0.0038 (8)	0.0046 (8)	0.0017 (8)
C6	0.0402 (9)	0.0414 (8)	0.0402 (8)	−0.0005 (7)	0.0049 (7)	0.0012 (7)
C7	0.0444 (10)	0.0406 (8)	0.0430 (9)	0.0029 (7)	0.0099 (7)	0.0004 (7)
C8	0.0436 (9)	0.0404 (8)	0.0377 (8)	0.0042 (7)	0.0053 (7)	−0.0011 (7)
C9	0.0463 (10)	0.0485 (10)	0.0463 (9)	−0.0020 (8)	0.0059 (7)	0.0018 (7)
C10	0.0526 (11)	0.0570 (11)	0.0417 (9)	0.0081 (8)	0.0122 (8)	0.0058 (8)
C11	0.0570 (11)	0.0579 (11)	0.0398 (9)	0.0073 (9)	0.0076 (8)	−0.0031 (8)
C12	0.0525 (11)	0.0515 (10)	0.0456 (10)	−0.0012 (8)	0.0044 (8)	−0.0080 (8)
C13	0.0436 (9)	0.0419 (8)	0.0381 (8)	0.0035 (7)	0.0055 (7)	−0.0005 (7)
C14	0.0485 (10)	0.0447 (9)	0.0374 (8)	0.0026 (8)	0.0016 (7)	−0.0030 (7)
C15	0.0411 (9)	0.0371 (8)	0.0402 (8)	0.0034 (7)	0.0003 (7)	−0.0027 (6)
C16	0.0473 (10)	0.0490 (9)	0.0431 (9)	0.0009 (8)	−0.0026 (8)	−0.0052 (7)
C17	0.0414 (10)	0.0482 (10)	0.0590 (11)	−0.0040 (8)	0.0007 (8)	−0.0057 (8)
C18	0.0480 (10)	0.0397 (9)	0.0541 (10)	0.0028 (7)	0.0120 (8)	0.0009 (7)
C19	0.0559 (11)	0.0537 (10)	0.0375 (9)	−0.0031 (8)	0.0017 (8)	−0.0001 (7)
C20	0.0429 (9)	0.0421 (9)	0.0432 (9)	−0.0017 (7)	−0.0009 (7)	−0.0033 (7)
C21	0.1081 (18)	0.0731 (14)	0.0560 (12)	0.0039 (13)	0.0201 (12)	−0.0142 (10)
C22	0.0542 (12)	0.0735 (14)	0.0982 (17)	−0.0124 (10)	0.0209 (11)	0.0092 (12)
C23	0.0717 (14)	0.0888 (15)	0.0553 (11)	−0.0013 (12)	0.0244 (10)	0.0064 (10)
C24	0.0886 (16)	0.0914 (16)	0.0512 (11)	0.0007 (13)	0.0195 (11)	−0.0204 (11)

### Geometric parameters (Å, °)

**Table:**

O1—C1	1.3489 (19)	C10—C23	1.515 (2)
O1—H1	0.9496	C11—C12	1.387 (2)
O2—C20	1.3532 (19)	C11—C24	1.514 (2)
O2—H2	0.9553	C12—C13	1.392 (2)
O3—C3	1.364 (2)	C12—H12A	0.9300
O3—C21	1.412 (2)	C14—C15	1.437 (2)
O4—C18	1.3678 (19)	C14—H14A	0.9300
O4—C22	1.430 (2)	C15—C16	1.393 (2)
N1—C7	1.2798 (19)	C15—C20	1.411 (2)
N1—C8	1.4142 (19)	C16—C17	1.379 (2)
N2—C14	1.281 (2)	C16—H16A	0.9300
N2—C13	1.412 (2)	C17—C18	1.384 (2)
C1—C2	1.394 (2)	C17—H17A	0.9300
C1—C6	1.402 (2)	C18—C19	1.385 (2)
C2—C3	1.383 (2)	C19—C20	1.377 (2)
C2—H2A	0.9300	C19—H19A	0.9300
C3—C4	1.389 (3)	C21—H21A	0.9600
C4—C5	1.361 (2)	C21—H21B	0.9600
C4—H4A	0.9300	C21—H21C	0.9600
C5—C6	1.397 (2)	C22—H22A	0.9600
C5—H5A	0.9300	C22—H22B	0.9600
C6—C7	1.442 (2)	C22—H22C	0.9600
C7—H7A	0.9300	C23—H23A	0.9600
C8—C9	1.391 (2)	C23—H23B	0.9600
C8—C13	1.395 (2)	C23—H23C	0.9600
C9—C10	1.387 (2)	C24—H24A	0.9600
C9—H9A	0.9300	C24—H24B	0.9600
C10—C11	1.390 (2)	C24—H24C	0.9600
			
C1—O1—H1	110.0	N2—C14—C15	121.61 (15)
C20—O2—H2	106.0	N2—C14—H14A	119.2
C3—O3—C21	118.90 (16)	C15—C14—H14A	119.2
C18—O4—C22	118.07 (15)	C16—C15—C20	117.59 (15)
C7—N1—C8	119.49 (13)	C16—C15—C14	121.04 (15)
C14—N2—C13	123.01 (14)	C20—C15—C14	121.37 (15)
O1—C1—C2	118.58 (14)	C17—C16—C15	122.47 (16)
O1—C1—C6	120.76 (14)	C17—C16—H16A	118.8
C2—C1—C6	120.65 (15)	C15—C16—H16A	118.8
C3—C2—C1	119.35 (16)	C16—C17—C18	118.49 (16)
C3—C2—H2A	120.3	C16—C17—H17A	120.8
C1—C2—H2A	120.3	C18—C17—H17A	120.8
O3—C3—C2	124.51 (17)	O4—C18—C17	124.02 (16)
O3—C3—C4	114.88 (17)	O4—C18—C19	115.09 (15)
C2—C3—C4	120.61 (16)	C17—C18—C19	120.88 (15)
C5—C4—C3	119.56 (17)	C20—C19—C18	120.23 (15)
C5—C4—H4A	120.2	C20—C19—H19A	119.9
C3—C4—H4A	120.2	C18—C19—H19A	119.9
C4—C5—C6	121.98 (16)	O2—C20—C19	118.56 (15)
C4—C5—H5A	119.0	O2—C20—C15	121.14 (14)
C6—C5—H5A	119.0	C19—C20—C15	120.31 (15)
C5—C6—C1	117.82 (14)	O3—C21—H21A	109.5
C5—C6—C7	120.37 (15)	O3—C21—H21B	109.5
C1—C6—C7	121.80 (14)	H21A—C21—H21B	109.5
N1—C7—C6	123.11 (14)	O3—C21—H21C	109.5
N1—C7—H7A	118.4	H21A—C21—H21C	109.5
C6—C7—H7A	118.4	H21B—C21—H21C	109.5
C9—C8—C13	118.80 (14)	O4—C22—H22A	109.5
C9—C8—N1	122.38 (15)	O4—C22—H22B	109.5
C13—C8—N1	118.81 (13)	H22A—C22—H22B	109.5
C10—C9—C8	122.33 (16)	O4—C22—H22C	109.5
C10—C9—H9A	118.8	H22A—C22—H22C	109.5
C8—C9—H9A	118.8	H22B—C22—H22C	109.5
C9—C10—C11	119.02 (15)	C10—C23—H23A	109.5
C9—C10—C23	119.55 (17)	C10—C23—H23B	109.5
C11—C10—C23	121.43 (16)	H23A—C23—H23B	109.5
C12—C11—C10	118.72 (15)	C10—C23—H23C	109.5
C12—C11—C24	119.12 (17)	H23A—C23—H23C	109.5
C10—C11—C24	122.16 (16)	H23B—C23—H23C	109.5
C11—C12—C13	122.60 (16)	C11—C24—H24A	109.5
C11—C12—H12A	118.7	C11—C24—H24B	109.5
C13—C12—H12A	118.7	H24A—C24—H24B	109.5
C12—C13—C8	118.50 (14)	C11—C24—H24C	109.5
C12—C13—N2	124.33 (15)	H24A—C24—H24C	109.5
C8—C13—N2	116.99 (13)	H24B—C24—H24C	109.5
			
O1—C1—C2—C3	−179.53 (15)	C10—C11—C12—C13	−0.3 (3)
C6—C1—C2—C3	−0.1 (2)	C24—C11—C12—C13	179.54 (17)
C21—O3—C3—C2	−1.4 (3)	C11—C12—C13—C8	−0.9 (3)
C21—O3—C3—C4	179.18 (18)	C11—C12—C13—N2	−175.78 (16)
C1—C2—C3—O3	−178.09 (16)	C9—C8—C13—C12	2.1 (2)
C1—C2—C3—C4	1.3 (3)	N1—C8—C13—C12	−178.65 (15)
O3—C3—C4—C5	177.60 (17)	C9—C8—C13—N2	177.33 (14)
C2—C3—C4—C5	−1.8 (3)	N1—C8—C13—N2	−3.4 (2)
C3—C4—C5—C6	1.2 (3)	C14—N2—C13—C12	−19.3 (2)
C4—C5—C6—C1	0.0 (3)	C14—N2—C13—C8	165.74 (15)
C4—C5—C6—C7	179.05 (17)	C13—N2—C14—C15	175.71 (14)
O1—C1—C6—C5	178.89 (15)	N2—C14—C15—C16	179.45 (15)
C2—C1—C6—C5	−0.6 (2)	N2—C14—C15—C20	−0.8 (2)
O1—C1—C6—C7	−0.1 (2)	C20—C15—C16—C17	−1.4 (2)
C2—C1—C6—C7	−179.59 (15)	C14—C15—C16—C17	178.33 (15)
C8—N1—C7—C6	179.24 (14)	C15—C16—C17—C18	0.0 (3)
C5—C6—C7—N1	177.82 (15)	C22—O4—C18—C17	1.5 (2)
C1—C6—C7—N1	−3.2 (2)	C22—O4—C18—C19	−177.94 (16)
C7—N1—C8—C9	−45.6 (2)	C16—C17—C18—O4	−177.89 (15)
C7—N1—C8—C13	135.15 (16)	C16—C17—C18—C19	1.5 (3)
C13—C8—C9—C10	−2.2 (2)	O4—C18—C19—C20	177.83 (15)
N1—C8—C9—C10	178.57 (15)	C17—C18—C19—C20	−1.6 (3)
C8—C9—C10—C11	1.0 (3)	C18—C19—C20—O2	179.93 (15)
C8—C9—C10—C23	−178.04 (16)	C18—C19—C20—C15	0.2 (2)
C9—C10—C11—C12	0.2 (3)	C16—C15—C20—O2	−178.47 (14)
C23—C10—C11—C12	179.28 (17)	C14—C15—C20—O2	1.8 (2)
C9—C10—C11—C24	−179.56 (18)	C16—C15—C20—C19	1.3 (2)
C23—C10—C11—C24	−0.5 (3)	C14—C15—C20—C19	−178.46 (15)

### Hydrogen-bond geometry (Å, °)

**Table:**

*D*—H···*A*	*D*—H	H···*A*	*D*···*A*	*D*—H···*A*
O1—H1···N1	0.95	1.78	2.6158 (18)	145
O2—H2···N2	0.96	1.71	2.5791 (17)	150
